# AS1411 Aptamer-Conjugated Liposomal siRNA Targeting MTA2 Suppresses PI3K/AKT Signaling in Pancreatic Cancer Cells

**DOI:** 10.3390/ijms26178467

**Published:** 2025-08-30

**Authors:** Minseo Kwak, Truong Chinh Hua, Hyesoo Jin, Jongsam Lee, Dong-Eun Kim

**Affiliations:** Department of Bioscience and Biotechnology, Konkuk University, Seoul 05029, Republic of Korea; zzanga0823@konkuk.ac.kr (M.K.); truongchinh.humus@gmail.com (T.C.H.); il_mondo00@naver.com (H.J.); znznfn2378@naver.com (J.L.)

**Keywords:** AS1411 aptamer, liposome, MTA2 siRNA, targeted delivery, pancreatic ductal adenocarcinoma, PI3K/AKT signaling

## Abstract

Pancreatic ductal adenocarcinoma (PDAC) is among the most lethal malignancies due to late diagnosis, poor drug penetration, and intrinsic chemoresistance. Targeted delivery strategies are urgently needed to enhance therapeutic precision while minimizing systemic toxicity. Here, we developed an AS1411 aptamer-functionalized liposomal platform encapsulating siRNA against metastasis-associated protein 2 (MTA2), a chromatin remodeling factor that suppresses the tumor suppressor PTEN and activates PI3K/AKT signaling. The AS1411 aptamer, which binds nucleolin overexpressed on PDAC cells, was conjugated to cationic liposomes via copper-free click chemistry. The resulting AS1411-Lipm[siRNA] exhibited high siRNA encapsulation efficiency, selective uptake by nucleolin-positive PDAC cells, and enhanced endosomal escape. Treatment of MIA PaCa-2 cells with AS1411-Lipm[siRNA] significantly reduced MTA2 expression by ~60%, substantially restored PTEN, and inhibited AKT phosphorylation by ~50%, leading to decreased cell viability, impaired migration by ~75%, and increased apoptosis by ~35%, while sparing nucleolin-negative cells. These findings highlight AS1411-Lipm[siRNA] as a promising platform for selective siRNA delivery and potent molecular inhibition in PDAC therapy.

## 1. Introduction

Pancreatic ductal adenocarcinoma (PDAC) is a highly aggressive malignancy in which hyperactivation of the PI3K/AKT signaling pathway—often driven by epigenetic or genetic loss of the tumor suppressor phosphatase and tensin homolog (PTEN)—contributes to rapid progression, therapeutic resistance, and poor survival outcomes [1,2,3]. PDAC is expected to account for the second leading cause of cancer-related mortality by 2030, with a 5-year survival rate under 13% [4,5,6]. This dismal prognosis stems from its late diagnosis, early metastatic spread, and limited efficacy of conventional treatments such as surgery, chemotherapy, and radiotherapy [7,8]. The systemic toxicity, low tumor specificity, and frequent emergence of resistance to these modalities underscore the critical requirement for novel strategies that can selectively control oncogenic signaling in PDAC [9,10].

RNA interference (RNAi) offers a powerful approach for gene-specific silencing in cancer therapy [11,12]. Small interfering RNA (siRNA) can precisely downregulate oncogenic drivers, creating a therapeutic opportunity in genetically defined malignancies such as PDAC [13,14]. Among emerging targets, metastasis-associated protein 2 (MTA2) is overexpressed in PDAC and promotes tumor progression partly by repressing PTEN transcription, thereby fueling PI3K/AKT hyperactivation [15,16]. Silencing MTA2 via siRNA could therefore suppress metastatic signaling while restoring tumor-suppressive functions in PDAC [15].

However, clinical translation of siRNA remains hindered by poor stability in circulation, limited tumor penetration, inefficient endosomal escape, and rapid renal clearance [17,18]. To overcome these barriers, nanocarrier systems—particularly liposomes—have been widely explored for siRNA delivery [19,20]. Liposomes can be engineered with cationic or ionizable lipids for siRNA complexation, helper lipids (e.g., cholesterol, DOPE) for membrane stability, and PEGylated lipids for extended circulation [19,20,21]. They protect siRNA from nuclease degradation, facilitate cellular uptake, and can be designed for stimuli-responsive release [22]. Notably, several liposomal siRNA formulations have advanced to clinical translation, validating their feasibility [23]. Nevertheless, conventional liposomes often rely on the enhanced permeability and retention (EPR) effect for tumor accumulation, which is severely limited in PDAC due to dense desmoplastic stroma, poor vascularization, and elevated interstitial fluid pressure [24,25]. This limitation highlights the need for active targeting strategies to ensure efficient and specific siRNA delivery to PDAC cells [26].

Aptamers are single-stranded nucleic acids that are able to form unique three-dimensional conformation, enabling high-affinity and high-specificity binding to molecular targets [27,28]. They are non-immunogenic, chemically synthesizable, and readily modifiable, serving as ideal ligands for targeted drug delivery [27,28]. Among these, the G-quadruplex-structured DNA aptamer AS1411 binds with strong affinity to nucleolin, which is a protein abundantly present on the surface of various tumor cells, including PDAC, but minimally expressed in normal tissues [29,30]. AS1411 has thus been widely employed as a tumor-targeting scaffold in nanoparticle-based delivery systems [31,32].

Our research group has extensively developed aptamer-conjugated liposomal systems for targeted cancer therapy. We previously demonstrated that RNA aptamer-functionalized liposomes could efficiently deliver chemotherapeutics to tumor sites in vivo, achieving enhanced tumor accumulation and therapeutic efficacy [33]. We further advanced this strategy by engineering dual-aptamer-conjugated liposomes targeting both MUC1 and CD44, enabling selective delivery to breast cancer cells and cancer stem cells [34]. More recently, we developed aptamer-conjugated nanoliposomes for immunogenic chemotherapy capable of reversing tumor-associated immunosuppression [35]. These studies underscore the versatility and translational potential of aptamer–liposome platforms, providing a strong rationale for applying AS1411-functionalized liposomes for targeted siRNA delivery in PDAC.

In this work, we developed an AS1411-conjugated liposomal siRNA delivery system (AS1411-Lipm[siRNA]) designed to silence MTA2 in nucleolin-overexpressing PDAC cells. The AS1411 aptamer was covalently linked to the liposomal surface via copper-free click chemistry under mild conditions, ensuring structural integrity and targeting capability. This platform achieved high siRNA encapsulation efficiency, selective cellular uptake, and efficient gene silencing in vitro. Our findings highlight AS1411-Lipm[siRNA] as a promising targeted gene therapy strategy for PDAC and potentially other nucleolin-positive malignancies.

## 2. Results

### 2.1. Screening and Selection of the Optimal Aptamer for PDAC-Targeted Delivery

**Figure 1:**
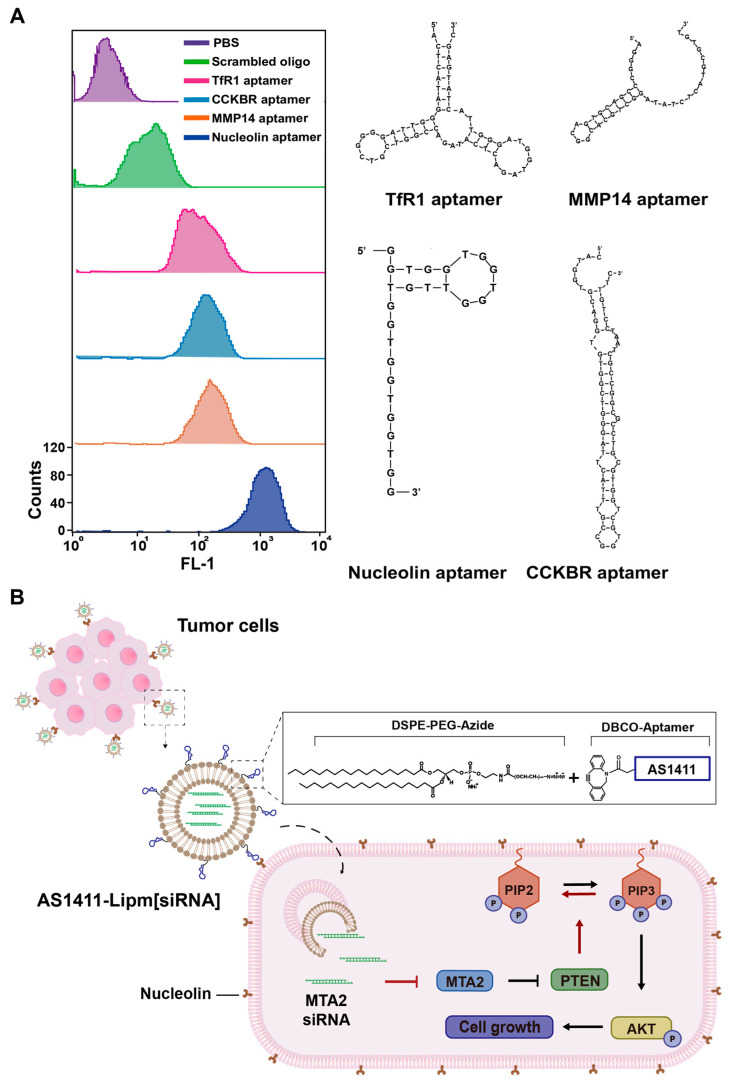
Structures of selected aptamers, comparative cellular uptake in pancreatic cancer cells, and schematic of the AS1411-Lipm[siRNA] platform. (**A**) Predicted secondary structures of anti-TfR1 (XQ-2D), anti-MMP14 (M17), anti-CCKBR (AP1153), and anti-nucleolin (AS1411) aptamers, and their intracellular uptake in MIA PaCa-2 cells. Cells were incubated with FAM-labeled aptamers for 4 h, and fluorescence intensity was analyzed by flow cytometry (FL-1 channel). Histogram overlays indicate relative uptake efficiency. (**B**) Schematic representation of the AS1411-conjugated liposomal delivery system encapsulating MTA2 siRNA. The platform is designed to target nucleolin-overexpressing PDAC cells, enabling selective gene silencing and induction of apoptosis through modulation of downstream oncogenic pathways.

To identify the most effective DNA aptamer for facilitating targeted uptake in pancreatic cancer cells, four candidates were selected based on their reported high affinities for tumor-associated surface proteins: AS1411 (anti-nucleolin), XQ-2D (anti-transferrin receptor 1), AP1153 (anti-cholecystokinin B receptor), and M17 (anti-matrix metalloproteinase 14) (Figure 1A, Table 1).

MIA PaCa-2 cells were incubated with 3′-FAM-labeled aptamers for 4 h, and intracellular fluorescence was quantified by flow cytometry. Among the tested sequences, AS1411 yielded the highest fluorescence signal, indicating the most efficient cellular internalization. Based on its superior uptake efficiency, AS1411 was selected as the targeting ligand for subsequent liposomal siRNA carrier design.

Building upon our previous work on aptamer-functionalized liposomes and lipid nanoparticles for cancer-targeted delivery [33,34,35], we developed an AS1411-conjugated liposomal siRNA platform (AS1411-Lipm[siRNA]) aimed at selectively silencing MTA2 in nucleolin-overexpressing pancreatic ductal adenocarcinoma (PDAC) cells, a malignancy highly resistant to conventional therapy (Figure 1B).

**Table 1:** Representative aptamers targeting cancer-associated cell-surface receptors and their reported binding affinities.

Name	Target	Sequence (5′-3′)	^1^ K_d_ (nM)	Reference
AS1411	Nucleolin	GGTGGTGGTGGTG-TTGGTGGTGGTGG	16.36 ± 10.30	[36]
M17	**^2^** MMP14	AGGGCCCGACGTGACGGCACGTCGGATATCTCATGCGTGT	4.98 ± 1.26	[37]
XQ-2D	^3^ TfR1	ACTCATAGGGTTAGGGGCTGCT-GGCCAGATACTCAGATGGTAGGGTTACTATGAGC	55.02 ± 0.4	[38]
AP1153	^4^ CCKBR	CATGGTGCAGGTGTGGCTGGGATTCATTTGCCGGTGCTGGTGCGTCCGCGG-CCGCTAATCCTGTTC	0.206	[39]

^1^ K_d_: Dissociation constant; ^2^ MMP14: Matrix metalloproteinase 14; ^3^ TfR1: Transferrin receptor 1; ^4^ CCKBR: Cholecystokinin B receptor.

### 2.2. Preparation and Physicochemical Characterization of AS1411-Functionalized Liposomal siRNA Formulations

To establish conditions for stable aptamer display, the conjugation of AS1411 to azide-functionalized lipid micelles was first optimized (Figure S1). Among the tested molar ratios of DBCO-modified AS1411 to micelles, a 1:96 ratio resulted in the complete disappearance of unreacted aptamer bands in agarose gel electrophoresis, indicating efficient coupling. siRNA encapsulation was performed using cationic liposomes consisting of DOTAP, DOPE, cholesterol, and PEG-DSPE2000, hydrated with siRNA at a nitrogen-to-phosphate (N/P) ratio of 4:1. DOTAP was chosen for strong cationic siRNA complexation, cholesterol for membrane rigidity, DOPE for promoting endosomal escape, and DSPE-PEG2000 for steric stabilization and circulation half-life. Encapsulation efficiency was confirmed by agarose gel electrophoresis, showing complete siRNA retention in the absence of detergent and full release upon Triton X-100 treatment (Figure S2).

**Figure 2:**
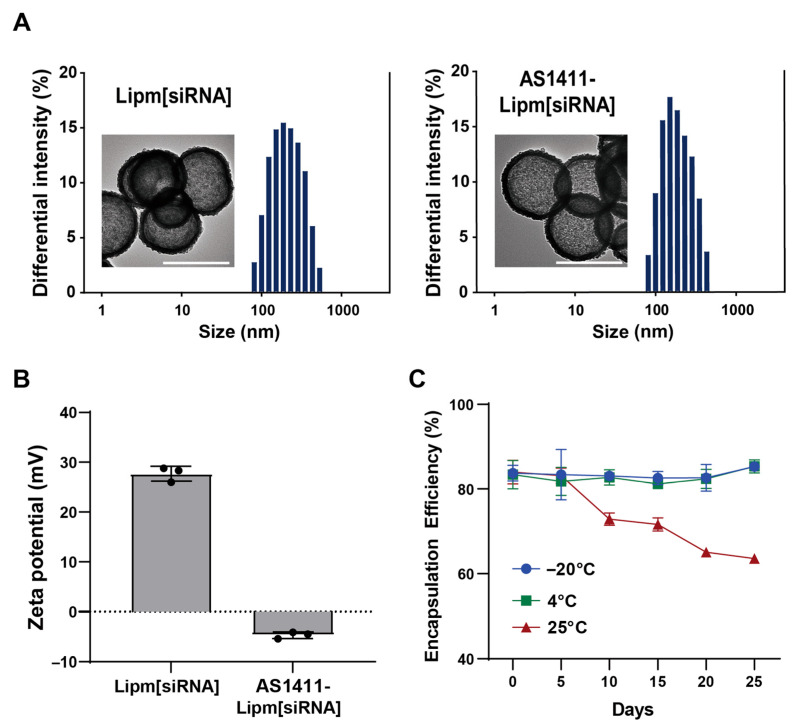
Physicochemical characterization and storage stability of AS1411-functionalized liposomal siRNA formulations. (**A**) Particle size profiles determined by dynamic light scattering (DLS) and morphological features observed by transmission electron microscopy (TEM) for Lipm[siRNA] and AS1411-Lipm[siRNA]. Scale bar = 200 nm. (**B**) Zeta potential measurements comparing Lipm[siRNA] and AS1411-Lipm[siRNA], illustrating surface charge alterations following AS1411 conjugation. (**C**) Stability of siRNA encapsulation in AS1411-Lipm[siRNA] kept at −20 °C, 4 °C, or 25 °C over 25 days, assessed by encapsulation efficiency using the RiboGreen assay.

For targeted delivery, AS1411-conjugated micelles were inserted post-synthetically into preformed siRNA-encapsulated liposomes at a 3:1 volume ratio, yielding the final AS1411-Lipm[siRNA] construct. Morphological stability following aptamer incorporation was assessed by transmission electron microscopy, which demonstrated that all liposomal formulations retained spherical vesicle structure without evidence of aggregation (Figure 2A). Next, particle size was measured to determine whether AS1411 conjugation altered the hydrodynamic diameter of liposomal formulations (Table 2). Dynamic light scattering revealed that size increased from 138.2 ± 3.56 nm (Lipm) and 136.4 ± 6.60 nm (Lipm[siRNA]) to 183.0 ± 0.51 nm (AS1411-Lipm) and 185.9 ± 24.8 nm (AS1411-Lipm[siRNA]). All liposomal formulations exhibited a monodisperse profile, as evidenced by polydispersity index (PDI) values remaining under 0.2.

For targeted delivery, AS1411-conjugated micelles were post-synthetically inserted into preformed siRNA-loaded liposomes at a 3:1 volume ratio, yielding the final AS1411-Lipm[siRNA] construct. Transmission electron microscopy demonstrated that all formulations maintained spherical morphology without aggregation following aptamer incorporation (Figure 2A). Dynamic light scattering revealed an increase in hydrodynamic diameter from 138.2 ± 3.56 nm (Lipm) and 136.4 ± 6.60 nm (Lipm[siRNA]) to 183.0 ± 0.51 nm (AS1411-Lipm) and 185.9 ± 24.8 nm (AS1411-Lipm[siRNA]) (Table 2). All formulations exhibited monodisperse profiles with polydispersity index (PDI) values below 0.2.

Surface charge measurements showed that non-targeted liposomes (Lipm and Lipm[siRNA]) possessed strongly cationic zeta potentials of +27.1 ± 0.64 mV and +27.5 ± 1.10 mV, respectively. In contrast, AS1411-functionalized liposomes (AS1411-Lipm and AS1411-Lipm[siRNA]) exhibited near-neutral charges (−2.66 ± 0.99 mV and −4.71 ± 0.42 mV), consistent with successful presentation of the negatively charged aptamer on the liposomal surface (Figure 2B, Table 2). Stability assays demonstrated that formulations stored at −20 °C or 4 °C retained over 80% of their encapsulated siRNA after 25 days, whereas storage at 25 °C resulted in a gradual decline to ~65% retention (Figure 2C).

**Table 2:** Physicochemical parameters of liposomal formulations with and without AS1411 conjugation and siRNA encapsulation.

Formulation	Particle Diameter (nM)	^1^ PDI	Zeta Potential (mV)
Lipm	138.2 ± 3.56	0.164 ± 0.001	27.3 ± 0.68
AS1411-Lipm	183.0 ± 0.513	0.171 ± 0.001	−2.66 ± 2.33
Lipm[siRNA]	136.4 ± 6.60	0.168 ± 0.012	27.7 ± 1.49
AS1411-Lipm[siRNA]	185.9 ± 24.8	0.164 ± 0.016	−4.71 ± 0.66

^1^ PDI: polydispersity index.

### 2.3. AS1411-Mediated Targeting Efficiency in Pancreatic Cancer Cells

To confirm nucleolin as a viable target for selective delivery, nucleolin expression was compared between MIA PaCa-2 pancreatic cancer cells and non-malignant L929 fibroblasts (Figure S3). Western blot analysis revealed markedly elevated nucleolin levels in MIA PaCa-2 cells, whereas L929 cells exhibited only minimal expression. Based on these findings, MIA PaCa-2 was assigned as nucleolin-positive, with L929 serving as the nucleolin-negative counterpart.

**Figure 3:**
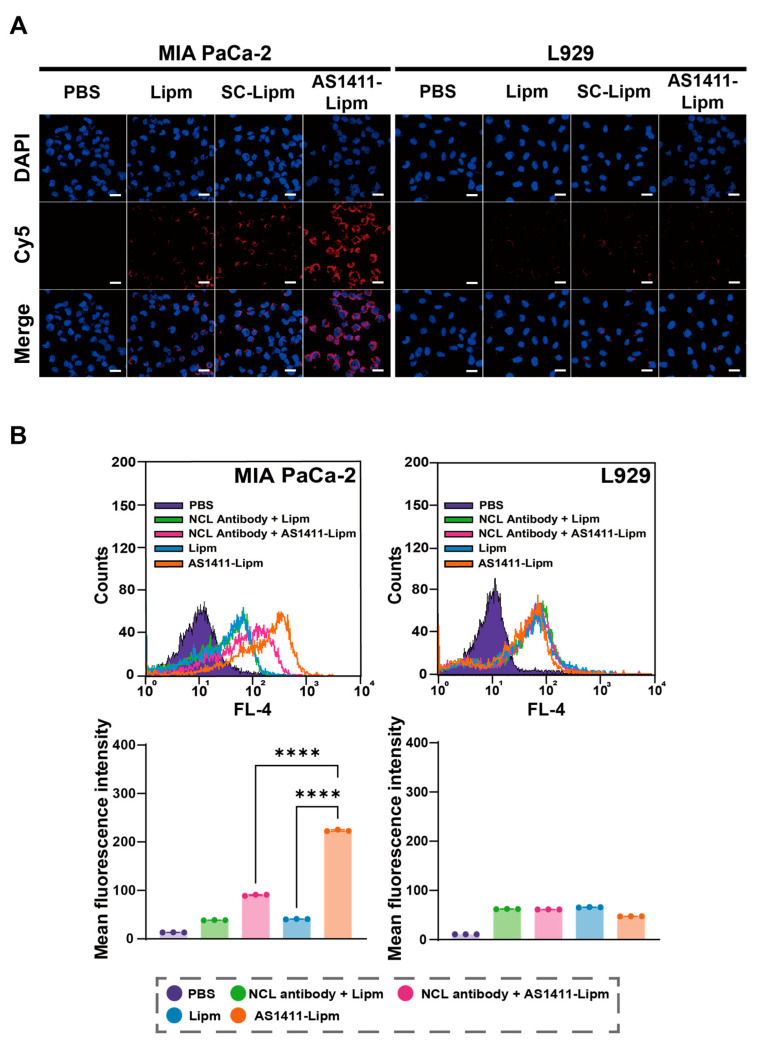
AS1411 aptamer promotes nucleolin-dependent uptake of liposomes in pancreatic cancer cells. (**A**) Confocal fluorescence images of MIA PaCa-2 (nucleolin-positive) and L929 (nucleolin-negative) cells incubated for 40 min with Cy5-labeled non-targeted liposomes (Lipm), scrambled aptamer-conjugated liposomes (SC-Lipm), or AS1411-conjugated liposomes (AS1411-Lipm). Liposomes are shown in red (Cy5) and nuclei in blue (DAPI). Scale bar = 20 μm. (**B**) Flow cytometry analysis of Cy5-labeled liposome uptake in cells pretreated or not with an anti-nucleolin (NCL) antibody. Upper panels: representative fluorescence histograms. Lower panels: mean fluorescence intensity (MFI) values for MIA PaCa-2 and L929 cells. **** *p* < 0.0001 compared with non-blocked AS1411-Lipm[siRNA] in MIA PaCa-2 cells.

The targeting specificity of AS1411-functionalized liposomes was evaluated by assessing their cellular uptake in these models (Figure 3A). Cells were incubated with Cy5-labeled non-targeted liposomes (Lipm), scrambled aptamer-conjugated liposomes (SC-Lipm), or AS1411-conjugated liposomes (AS1411-Lipm) for 40 min at 37 °C, followed by confocal fluorescence imaging. In MIA PaCa-2 cells, AS1411-Lipm exhibited substantially stronger red fluorescence compared with Lipm or SC-Lipm, indicating enhanced internalization. In contrast, fluorescence intensity among the three formulations was comparable in L929 cells, suggesting minimal non-specific uptake.

To verify nucleolin-dependent binding, competitive inhibition experiments were performed using an anti-nucleolin antibody (Figure 3B). Pre-incubation of MIA PaCa-2 cells with the blocking antibody significantly reduced Cy5 fluorescence from AS1411-Lipm[siRNA], whereas Lipm uptake remained unaffected. No significant changes were observed in L929 cells under any condition. These results confirm that AS1411-conjugated liposomes undergo selective uptake in nucleolin-overexpressing pancreatic cancer cells through a nucleolin-mediated mechanism.

### 2.4. AS1411-Conjugated Liposomes Enhance Targeted siRNA Uptake and Promote Endosomal Escape in Nucleolin-Positive Pancreatic Cancer Cells

**Figure 4:**
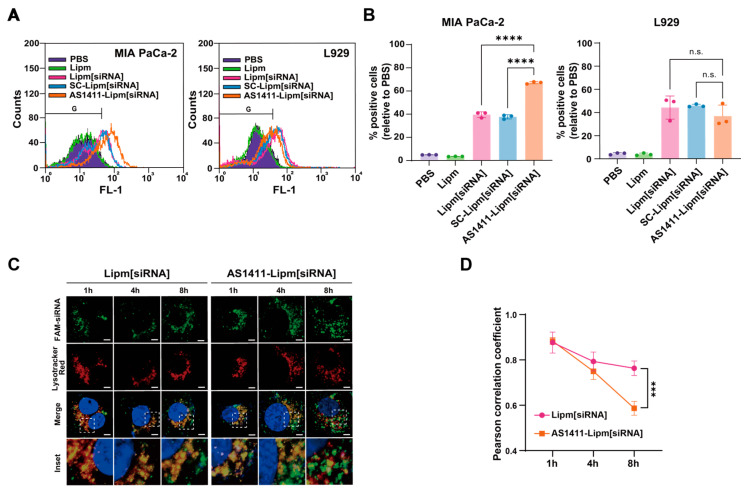
Selective siRNA delivery and enhanced endosomal escape mediated by AS1411-conjugated liposomes in pancreatic cancer cells. (**A**) Flow cytometry histograms showing uptake of FAM-labeled siRNA in MIA PaCa-2 (nucleolin-positive) and L929 (nucleolin-negative) cells after 30 min incubation with PBS, non-targeted liposomes (Lipm), siRNA-loaded liposomes (Lipm[siRNA]), scrambled aptamer-conjugated siRNA-loaded liposomes (SC-Lipm[siRNA]), or AS1411-conjugated siRNA-loaded liposomes (AS1411-Lipm[siRNA]); the gating threshold (G) was defined as the upper 5% fluorescence of PBS-treated cells. (**B**) Quantification of FAM-positive cell populations exceeding the gating threshold from (**A**); **** *p* < 0.0001; n.s., not significant. (**C**) Confocal microscopy images of MIA PaCa-2 cells treated with Lipm[siRNA] or AS1411-Lipm[siRNA], showing FAM-siRNA (green), endo-/lysosomes labeled with LysoTracker Red (red), and nuclei stained with 4′,6-diamidino-2-phenylindole (DAPI; blue); scale bar = 5 μm. (**D**) Pearson correlation coefficients between FAM-siRNA and LysoTracker signals at 1, 4, and 8 h post-treatment, calculated using ImageJ software (version 1.54p); *** *p* < 0.001.

To assess whether AS1411 aptamer functionalization enhances siRNA delivery, MIA PaCa-2 (nucleolin-positive) and L929 (nucleolin-negative) cells were treated with PBS, Lipm, Lipm[siRNA], SC-Lipm[siRNA], or AS1411-Lipm[siRNA] (Figure 4A). Flow cytometry revealed that AS1411-Lipm[siRNA] markedly increased the proportion of FAM-positive MIA PaCa-2 cells compared with all other groups. Quantitative gating analysis, using PBS-treated cells as baseline, confirmed a significant increase in siRNA-positive populations (Figure 4B). In contrast, L929 cells displayed no significant differences in uptake among liposomal groups, indicating that AS1411 conjugation confers selective delivery to nucleolin-expressing cells.

We next examined whether AS1411 functionalization facilitates cytosolic release of internalized siRNA. Confocal microscopy showed that, at early time points, siRNA delivered by both Lipm[siRNA] and AS1411-Lipm[siRNA] was predominantly colocalized with LysoTracker-positive compartments, consistent with endo-lysosomal entrapment (Figure 4C). By 8 h post-treatment, AS1411-Lipm[siRNA]-treated cells exhibited marked dissociation of FAM and LysoTracker signals, whereas Lipm[siRNA]-treated cells retained high colocalization. Quantification of Pearson correlation coefficients confirmed significantly lower colocalization in AS1411-Lipm[siRNA]-treated cells at 8 h (Figure 4D), indicating enhanced endosomal escape and greater availability of siRNA in the cytosol.

### 2.5. Targeted MTA2 Silencing via AS1411-Conjugated Liposomal siRNA Modulates PTEN Expression and PI3K/AKT Signaling

**Figure 5:**
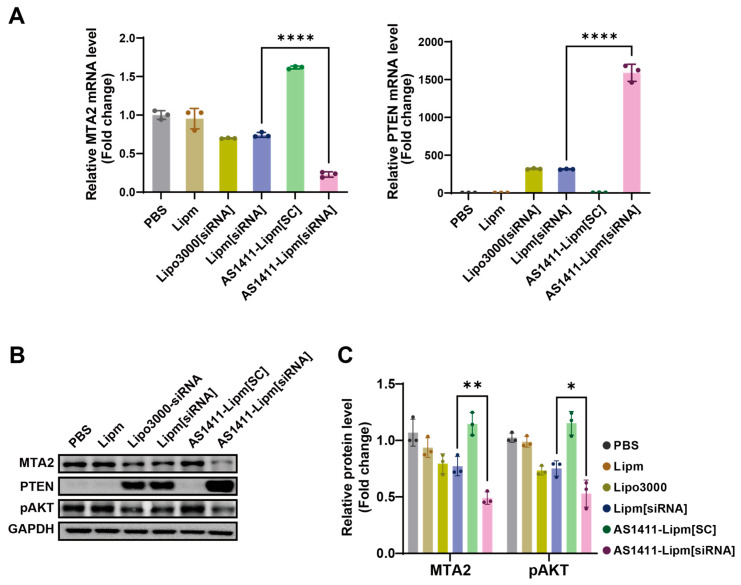
Targeted silencing of MTA2 and modulation of downstream signaling by AS1411-Lipm[siRNA] in MIA PaCa-2 cells. (**A**) Quantitative reverse transcription PCR (qRT-PCR) analysis of MTA2 and PTEN mRNA levels in MIA PaCa-2 cells following treatment with various liposomal formulations; mRNA levels were normalized to GAPDH as the internal reference gene and reported as fold change compared with PBS-treated controls; **** *p* < 0.0001. (**B**) Western blot analysis of MTA2, PTEN, and phosphorylated AKT (p-AKT) protein levels in MIA PaCa-2 cells following treatment with the same formulations. (**C**) Densitometric quantification of MTA2 and p-AKT protein expression from (**B**), measured by band intensity using ImageJ software and normalized to GAPDH; * *p* < 0.05, ** *p* < 0.01.

We next evaluated whether targeted siRNA delivery translated into gene silencing and pathway modulation. RT-qPCR analysis in MIA PaCa-2 cells showed that AS1411-Lipm[siRNA] significantly reduced MTA2 mRNA levels compared with non-targeted Lipm[siRNA] (Figure 5A). In parallel, PTEN mRNA was significantly upregulated, suggesting transcriptional activation as a downstream effect of MTA2 knockdown.

Protein analysis by Western blot demonstrated that AS1411-Lipm[siRNA] reduced MTA2 expression and increased PTEN abundance, consistent with the transcript data (Figure 5B). Moreover, phosphorylation of AKT at Ser473 was markedly decreased following AS1411-Lipm[siRNA] treatment, indicating suppression of PI3K/AKT signaling. Densitometric quantification confirmed these trends (Figure 5C), supporting that aptamer-guided delivery enhances siRNA potency and downstream tumor-suppressive effects.

### 2.6. Targeted Delivery of MTA2 siRNA via AS1411 Liposomes Elicits Potent Anticancer Effects in Pancreatic Cancer Cells

**Figure 6:**
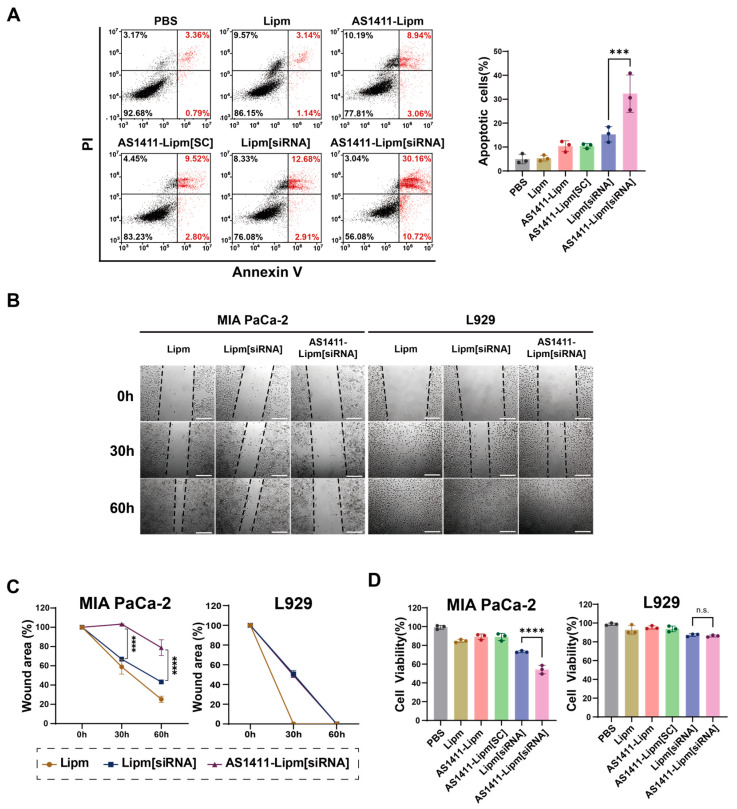
In vitro anticancer efficacy of AS1411-Lipm[siRNA] in pancreatic cancer cells. (**A**) Apoptosis induction in MIA PaCa-2 cells incubated with various liposomal formulations for 12 h, assessed via Annexin V–FITC/PI dual staining and examined by flow cytometry; dot plots show apoptotic cell populations, and bar graphs indicate the percentage of Annexin V^+^ cells; *** *p* < 0.001. (**B**) Wound healing assay in MIA PaCa-2 and L929 cells treated with the indicated formulations; representative phase-contrast micrographs were taken at 0, 30, and 60 h to monitor migration; scale bar = 275 μm. (**C**) Quantification of the wound healing assay from panel B; the percentage of remaining wound area at each time point was measured using ImageJ and normalized to the initial wound area at 0 h; **** *p* < 0.0001. (**D**) Cell viability measured by WST-1 assay in MIA PaCa-2 and L929 cells after 24 h treatment; viability is expressed relative to PBS-treated control; **** *p* < 0.0001; n.s., not significant.

The pro-apoptotic effect of AS1411-mediated MTA2 siRNA delivery was evaluated by quantifying apoptotic cell populations in MIA PaCa-2 cells following treatment with various liposomal formulations (Figure 6A). Apoptotic cells were analyzed through Annexin V–fluorescein isothiocyanate (FITC)/PI dual staining following 12 h treatment with PBS, Lipm, AS1411-Lipm, AS1411-Lipm[SC], Lipm[siRNA], or AS1411-Lipm[siRNA]. AS1411-Lipm[siRNA] treatment led to a notable rise in proportion of Annexin V-positive cells (32.34 ± 7.82%), nearly doubling that observed with Lipm[siRNA] (15.33 ± 3.21%) and exceeding all other groups, indicating enhanced apoptosis through selective siRNA delivery to nucleolin-positive cancer cells.

To determine whether the targeted delivery system impacts cell migration, wound healing assays were conducted in MIA PaCa-2 and L929 cells. After 12 h treatment with Lipm, Lipm[siRNA], or AS1411-Lipm[siRNA], wound closure was monitored at 0, 30, and 60 h (Figure 6B). In MIA PaCa-2 cells, AS1411-Lipm[siRNA] markedly delayed wound closure compared to the other two groups, indicating an inhibitory effect on cancer cell motility. By contrast, L929 cells displayed near-complete wound closure by 60 h across all groups, with only minor transient differences observed at 30 h. Quantification of the remaining wound area over time confirmed these trends (Figure 6C), supporting selective inhibition of migration in nucleolin-positive cells.

Overall cytotoxicity was further assessed by WST-1 assay in MIA PaCa-2 and L929 cells treated with each liposomal formulation for 24 h (Figure 6D). AS1411-Lipm[siRNA] treatment significantly reduced viability in MIA PaCa-2 cells compared to non-targeted formulations. In contrast, L929 cells exhibited similar viability across all treatments, indicating that the cytotoxic effect is largely dependent on nucleolin-specific targeting.

## 3. Discussion

In this study, we established a liposomal platform functionalized with the nucleolin-targeting aptamer AS1411 to enable selective delivery of siRNA into pancreatic cancer cells. Screening of candidate aptamers identified AS1411 as the most effective in binding nucleolin-overexpressing MIA PaCa-2 cells, leading us to select it as the targeting ligand. The final construct, AS1411-Lipm[siRNA], generated through azide–alkyne click chemistry and post-insertion, exhibited uniform particle size, stable surface charge, structural integrity, and high siRNA encapsulation efficiency, all of which are favorable attributes for further preclinical application. Moreover, TEM analysis indicated that AS1411-Lipm[siRNA] retained its morphology after 45 days of storage at 4 °C (Supplementary Figure S4), supporting its stability under these conditions. Nonetheless, additional studies under different storage temperatures and time points would be valuable to further substantiate the long-term stability profile of the formulation.

AS1411-Lipm[siRNA] displayed selective uptake in MIA PaCa-2 cells while showing negligible internalization in nucleolin-deficient L929 cells, supporting the role of aptamer recognition in driving cellular specificity. Once internalized, the construct promoted efficient cytosolic release of siRNA, addressing one of the key obstacles in RNA interference therapy. This combination of tumor-selective uptake and improved endosomal escape highlights the utility of aptamer-guided liposomes as reliable siRNA delivery vehicles.

Gene silencing studies confirmed that MTA2 expression was effectively reduced at both transcript and protein levels. Consequent restoration of PTEN expression and suppression of AKT phosphorylation disrupted the PI3K/AKT signaling pathway, indicating that the platform can modulate oncogenic signaling at multiple regulatory levels. Such molecular changes were accompanied by increased apoptosis, reduced proliferation, and diminished migration in MIA PaCa-2 cells, whereas L929 cells were minimally affected. These findings illustrate that the functional outcome of MTA2 silencing was selectively confined to nucleolin-positive tumor cells. Although the migration assay showed a clear reduction in wound closure (Figure 6B), part of this response may have arisen from the cytotoxic activity of the formulation. This possibility is consistent with the increased apoptotic population and reduced viability observed in parallel assays. Further studies assessing motility-related proteins or cell morphology would help to distinguish direct effects on migration from secondary consequences of reduced cell survival.

Importantly, the strategy demonstrated here is consistent with previous aptamer-guided delivery approaches that have been applied in other malignancies [39,40]. In addition, recent studies highlight that both aptamer-functionalized and liposome-based systems can be tailored either to remodel the tumor microenvironment or to achieve cell-specific uptake [41,42]. These studies support the versatility of aptamer-based therapeutics, while the present work extends this concept by showing that AS1411-conjugated liposomes effectively silence MTA2 and restore tumor-suppressive signaling specifically in PDAC.

The present work was conducted under 2D culture conditions, which allowed us to delineate cellular responses with precision. Nevertheless, three-dimensional spheroids and animal models will be important to confirm whether the observed selectivity and signaling modulation are preserved in more complex environments. Establishing such validation will further strengthen the translational value of AS1411-Lipm[siRNA] and define its potential in addressing the limitations of current PDAC therapies.

In conclusion, the AS1411-guided liposomal system presented here integrates molecular specificity, delivery stability, and enhanced cytosolic release to achieve selective MTA2 silencing. Through effective MTA2 inhibition and disruption of downstream oncogenic signaling, AS1411-Lipm[siRNA] demonstrates a strategy that directly addresses the challenges of siRNA-based cancer therapy and the broader limitations of PDAC treatment. These findings support continued development of aptamer-guided liposomal carriers as precise and versatile platforms for RNAi-based oncology applications.

## 4. Materials and Methods

### 4.1. Materials

The cationic lipid 1,2-dioleoyl-3-trimethylammonium-propane (DOTAP), helper lipids including cholesterol and 1,2-dioleoyl-sn-glycero-3-phosphoethanolamine (DOPE), 1,2-distearoyl-sn-glycero-3-phosphoethanolamine–polyethylene glycol 2000 (DSPE-PEG2000)-azide, and the fluorescent lipid Cy5-DOPE were purchased from Avanti Polar Lipids (Alabaster, AL, USA). DOTAP was chosen for strong cationic siRNA complexation, cholesterol for membrane rigidity, DOPE for promoting endosomal escape, and DSPE-PEG2000 for steric stabilization and circulation half-life. As a positive control for siRNA transfection, Lipofectamine™ 3000 (L3000015; Invitrogen, Carlsbad, CA, USA) was employed. Metastasis-associated protein 2 (MTA2)-specific siRNA (SI02780834) was obtained from QIAGEN (Hilden, Germany). Dibenzocyclooctyne (DBCO)-modified AS1411 DNA aptamer was synthesized by BIONICS (Daejeon, Republic of Korea). Liposome extrusion equipment was purchased from Avanti Polar Lipids, and polycarbonate membrane filters with pore sizes of 400, 200, and 100 nm were obtained from Whatman (Maidstone, UK).

For protein detection by Western blot, anti-PTEN (ab32199; Abcam, Cambridge, UK), anti-GAPDH (ab181602; Abcam), and anti-phospho-AKT (Ser473, 9271S; Cell Signaling Technology, Danvers, MA, USA) were applied as primary antibodies. Anti-nucleolin antibody (ZN004) was purchased from Invitrogen. MIA PaCa-2 (human pancreatic cancer) and L929 (mouse fibroblast) cell lines were obtained from the American Type Culture Collection (ATCC, Manassas, VA, USA). MIA PaCa-2 cells were grown in Dulbecco’s Modified Eagle Medium (DMEM, high glucose; Welgene, Gyeongsan, Republic of Korea), and L929 cells were propagated in Minimum Essential Medium (MEM; Biowest, Nuaillé, France). All culture media contained 10% fetal bovine serum and 1% penicillin–streptomycin as supplements. Cells were maintained at 37 °C in a humidified atmosphere containing 5% CO_2_.

### 4.2. Aptamer Screening via Flow Cytometry

Four candidate aptamers targeting transferrin receptor 1 (TfR1), cholecystokinin B receptor (CCKBR), matrix metalloproteinase-14 (MMP14), and nucleolin were synthesized with a 3′-end 6-carboxyfluorescein (6-FAM) modification (BIONICS, Daejeon, Republic of Korea). The sequences and corresponding names of these aptamers are summarized in Table 1. MIA PaCa-2 cells were seeded in 6-well dishes at a density of 3 × 10^5^ cells/well and incubated overnight under standard culture conditions (37 °C, 5% CO_2_, humidified atmosphere). Each aptamer was diluted in 200 μL of culture medium to a final concentration of 0.5 μM and applied to the cells the following day. Cells were incubated at 37 °C for 5 h, then washed with PBS, detached, and placed into PBS supplemented with 2% bovine serum albumin (BSA). Intracellular fluorescence was measured using a FACSCalibur™ flow cytometer (BD Biosciences, San Jose, CA, USA). All samples were analyzed under identical instrument settings to compare relative fluorescence intensities among aptamer-treated groups.

### 4.3. Preparation of AS1411-Conjugated Liposomal siRNA

#### 4.3.1. Preparation of AS1411-Aptamer-Conjugated Micelles

Azide-functionalized micelles were prepared by dissolving DSPE-PEG2000-azide and DSPE-PEG2000 in chloroform at a 4:1 molar ratio. Solvent removal was performed using nitrogen stream, and the obtained lipid film was further dried under vacuum conditions for no less than 1 h. The film was hydrated in HEPES buffer (20 mM HEPES, 150 mM NaCl, pH 7.4) and sonicated to facilitate micelle formation. For aptamer conjugation, DBCO-functionalized AS1411 was added to the micelle dispersion at a molar ratio of 1:96 (DBCO/azide) and incubated at 4 °C for 12 h to allow copper-free click chemistry between DBCO and azide groups.

#### 4.3.2. Preparation of siRNA-Encapsulated Cationic Liposomes

Cationic liposomes were formulated by mixing DOTAP, cholesterol, DOPE, and DSPE-PEG2000 at a molar ratio of 50:50:1:1 in chloroform. After removal of solvent under nitrogen gas, the lipid residue was desiccated in a vacuum chamber for an additional hour. The dried film was rehydrated with HEPES buffer containing MTA2 siRNA (20 mM HEPES, 150 mM NaCl, pH 7.4) at a nitrogen-to-phosphate (N/P) ratio of 4:1, followed by probe sonication for 5 min with vortexing at 1 min intervals. Liposomes were passed sequentially through polycarbonate membranes (400, 200, and 100 nm) to generate particles with uniform dimensions.

#### 4.3.3. Post-Insertion of Aptamer-Conjugated Micelles into Liposomes

AS1411-conjugated micelles were mixed with siRNA-loaded liposomes at a volume ratio of 1:3 (*v*/*v*) and incubated at 60 °C for 1 h to enable post-insertion of micelles into the liposomal bilayer. The mixture was then equilibrated at 25 °C for 1 h prior to subsequent experiments.

### 4.4. Physicochemical Characterization and Stability Evaluation

Liposomal physicochemical properties, including particle size, PDI, and ζ-potential, were analyzed using an ELS Z-1000 electrophoretic light-scattering system (Otsuka Electronics, Tokyo, Japan) after water dilution. Morphological evaluation was carried out by TEM (JEM-1010; JEOL, Tokyo, Japan). siRNA encapsulation was assessed by agarose gel electrophoresis. To evaluate protection of siRNA by the lipid bilayer, samples were either left intact or treated with Triton X-100 to disrupt the membrane, followed by analysis of electrophoretic mobility.

For storage stability assessment, siRNA-loaded liposomes were stored at −20 °C, 4 °C, or 25 °C for up to 25 days. At each time point, retained siRNA content was quantified using the Quant-iT™ RiboGreen™ RNA Assay Kit (R11490; Invitrogen, Carlsbad, CA, USA). Briefly, liposomes were diluted 1:25 in TE buffer and lysed with 20% Triton X-100 at 25 °C for 10 min. Samples were then incubated with 1% RiboGreen reagent, and fluorescence was measured using a VICTOR X3 microplate reader (PerkinElmer, Waltham, MA, USA) at an excitation wavelength of 485 nm and emission detection at 535 nm. Encapsulation efficiency (%) was calculated as follows: encapsulation efficiency (%) = (encapsulated siRNA [μg]/total siRNA input [μg]) × 100.

### 4.5. Evaluation of Intracellular Delivery Efficiency of Cy5-Labeled Liposomes

For confocal microscopy, MIA PaCa-2 and L929 cells were seeded on sterile coverslips in 24-well plates (5 × 10^4^ cells/well) and cultured overnight at 37 °C in a humidified incubator containing 5% CO_2_. Cells were treated with Cy5-labeled liposomes (30 μg/well) for 30 min at 37 °C, washed with PBS, and fixed with 4% paraformaldehyde for 30 min at room temperature. Nuclei were stained with DAPI (1:500; D1306, Invitrogen, Waltham, MA, USA), and coverslips were mounted with antifade reagent. Images were acquired using an LSM 800 confocal laser scanning microscope (Carl Zeiss, Oberkochen, Germany).

For flow cytometric analysis, MIA PaCa-2 cells were seeded in 6-well plates (3 × 10^5^ cells/well) and cultured overnight. To assess aptamer-mediated uptake specificity via nucleolin, cells were pre-incubated with anti-nucleolin antibody (1:1000 dilution, 2 h, 37 °C) prior to treatment with Cy5-labeled Lipm or AS1411-Lipm. Following a 30 min exposure to liposomes, cells were collected, rinsed with PBS, and resuspended in PBS supplemented with 2% bovine serum albumin (BSA). Fluorescence intensity was measured using a BD FACSCalibur™ flow cytometer (BD Biosciences, San Jose, CA, USA) with identical instrument settings for all samples.

### 4.6. Evaluation of siRNA Uptake Efficiency and Endosomal Escape Assay

For siRNA uptake analysis, MIA PaCa-2 cells were seeded in 6-well plates (3 × 10^5^ cells/well) and cultured overnight under standard conditions (37 °C, 5% CO_2_, humidified atmosphere). Cells were treated with Lipm, Lipm[siRNA], SC-Lipm[siRNA], or AS1411-Lipm[siRNA] for 40 min at 37 °C. Intracellular siRNA fluorescence was quantified by measuring FAM-siRNA signals using a BD FACSCalibur™ flow cytometer (BD Biosciences, San Jose, CA, USA).

For endosomal escape assessment, MIA PaCa-2 cells were seeded on sterile coverslips in 24-well plates (5 × 10^4^ cells/well) and incubated overnight. Cells were treated with Lipm[siRNA] or AS1411-Lipm[siRNA] (15 μg/mL) for 1, 4, or 8 h at 37 °C. LysoTracker™ Red DND-99 (250 nM; Life Technologies, Carlsbad, CA, USA) was added during the final 2 h of incubation to label lysosomes/endosomes. Following PBS washing, cells were fixed with 4% paraformaldehyde and stained with DAPI (1:500) for nuclear visualization. Images were captured using an LSM 800 confocal laser scanning microscope (Carl Zeiss, Oberkochen, Germany). Colocalization between FAM-siRNA and LysoTracker signals was analyzed qualitatively and quantitatively, with Pearson’s correlation coefficients calculated using ImageJ software (version 1.54p, National Institutes of Health, Bethesda, MD, USA) to evaluate endosomal escape efficiency.

### 4.7. Quantitative Reverse Transcription PCR (qRT-PCR) Analysis

To assess MTA2 and PTEN transcript levels, MIA PaCa-2 cells were treated with PBS, naked Lipm, Lipofectamine™ 3000 complexed with siRNA, Lipm[siRNA], SC-Lipm[siRNA], or AS1411-Lipm[siRNA] for 9 h at 37 °C. The culture medium was subsequently exchanged for fresh medium, and the cells were maintained for another 24 h. Total RNA was isolated with the TransZol-Up reagent (TransGen Biotech, Beijing, China) following the supplier’s protocol. cDNA was synthesized from 150 ng of total RNA in a 20 μL reaction using ReverTra Ace™ qPCR RT Master Mix (TOYOBO, Osaka, Japan).

qPCR analysis was executed on the StepOnePlus™ Real-Time PCR System using PowerUp™ SYBR™ Green Master Mix (Applied Biosystems, Foster City, CA, USA). The cycling conditions were as follows: 50 °C for 2 min, 95 °C for 2 min, followed by 40 cycles of 95 °C for 15 s and 60 °C for 1 min, with a melt curve stage of 95 °C for 15 s, 60 °C for 1 min, and 95 °C for 1 s. Primer sequences were as follows: MTA2: forward 5′-GCG CAG GGA CAT TTC TAG TAG-3′, reverse 5′-TGG GTG GCT GGT AAT GAT TCA-3′; PTEN: forward 5′-TTT GAA GAC CAT AAC CCA CCA C-3′, reverse 5′-ATT ACA CCA GTT CGT CCC TTT C-3′; GAPDH: forward 5′-ACC ACA GTC CAT GCC ATC AC-3′, reverse 5′-TCC ACC ACC CTG TTG CTG TA-3′. Relative mRNA expression was calculated using the 2^−ΔΔCt^ method, with GAPDH serving as the internal normalization control.

### 4.8. Western Blot Analysis

MIA PaCa-2 cells (3 × 10^5^ cells/well) were seeded in 6-well plates and cultured overnight at 37 °C in a humidified atmosphere containing 5% CO_2_. Cells were then treated with Lipm, AS1411-Lipm, AS1411-Lipm[SC], Lipm[siRNA], or AS1411-Lipm[siRNA] (2 μg/mL) for 9 h. Lipofectamine™ 3000 (Invitrogen, Carlsbad, CA, USA) was used as a positive transfection control. After treatment, cells were replaced with fresh medium and incubated for an additional 24 h.

Cell lysis was performed using RIPA buffer (Thermo Fisher Scientific, Waltham, MA, USA) containing both protease and phosphatase inhibitor cocktails. The concentration of proteins was measured employing a BCA Protein Assay Kit (Thermo Fisher Scientific). Proteins (15 μg per lane) were separated on 10% SDS–PAGE gels and subsequently transferred onto PVDF membranes (Millipore, Billerica, MA, USA). Membranes were blocked in 3% BSA in TBST (0.1% Tween-20 in Tris-buffered saline) for 1 h at room temperature, followed by overnight incubation at 4 °C with primary antibodies against MTA2, PTEN, phospho-AKT (Ser473), and GAPDH. After washing, HRP-conjugated secondary antibodies (Invitrogen) were applied for 2 h at room temperature.

Protein signals were detected by enhanced chemiluminescence (Thermo Fisher Scientific) and images were acquired with the G:BOX Chemi XL platform (Syngene, Cambridge, UK). Densitometric analysis of protein bands was performed with ImageJ software (NIH, Bethesda, MD, USA), with normalization to GAPDH as the internal loading control.

### 4.9. Apoptosis Assay

MIA PaCa-2 cells (3 × 10^5^ cells/well) were seeded in 6-well plates and cultured overnight at 37 °C in a humidified 5% CO_2_ atmosphere. Cells were then treated with PBS, Lipm, AS1411-Lipm, AS1411-Lipm[SC], Lipm[siRNA], or AS1411-Lipm[siRNA] (15 μg/mL) for 24 h. After treatment, cells were harvested, washed twice with ice-cold PBS, and resuspended in Annexin V–FITC/PI staining solution containing RNase A. Following a 15 min incubation in the dark at 25 °C, samples were analyzed by flow cytometry using a CytoFLEX system (Beckman Coulter, Brea, CA, USA). The proportion of cells undergoing early or late apoptosis was determined by analyzing fluorescence signal distribution.

### 4.10. Wound Healing Assay

MIA PaCa-2 cells (3 × 10^5^ cells/well) were seeded in 24-well plates and cultured overnight at 37 °C in a humidified 5% CO_2_ atmosphere. Cells were then treated with Lipm, Lipm[siRNA], or AS1411-Lipm[siRNA] (15 μg/mL) for 24 h. A straight wound was created across the confluent monolayer using a sterile pipette tip, and dislodged cells were eliminated by washing with PBS. Wound closure was monitored by capturing phase-contrast images at 0, 30, and 60 h using an EVOS M7000 microscope (Thermo Fisher Scientific, Waltham, MA, USA). The wound area was quantified at each time point using ImageJ software, and closure was expressed as the percentage of the remaining gap relative to the initial wound area.

### 4.11. Cell Viability Assay

MIA PaCa-2 cells (5 × 10^4^ cells/well) were seeded in 24-well plates and cultured for 12 h at 37 °C in a humidified 5% CO_2_ atmosphere. Cells were then treated with PBS, Lipm, AS1411-Lipm, Lipm[siRNA], AS1411-Lipm[SC], or AS1411-Lipm[siRNA] (15 μg/mL) for 9 h. Following treatment, cells were replaced with fresh medium and incubated for an additional 24 h under standard culture conditions. Cell viability measurements were performed with the WST-1 assay kit (DogenBio, Seoul, Republic of Korea) in accordance with the manufacturer’s guidelines. Measurement of absorbance at 450 nm was performed with a VICTOR X3 plate reader (PerkinElmer, Waltham, MA, USA), and results were presented as viability percentages compared to PBS-treated controls.

### 4.12. Statistical Analysis

All experiments were performed in triplicate (*n* = 3), and data are presented as mean ± standard deviation. Statistical analyses were conducted using GraphPad Prism 8 (GraphPad Software, San Diego, CA, USA). Multiple group comparisons were performed using one-way ANOVA followed by Dunnett’s multiple comparison test. A *p*-value < 0.05 was considered statistically significant. Significance levels are indicated as follows: * *p* < 0.05, ** *p* < 0.01, *** *p* < 0.001, **** *p* < 0.0001.

## 5. Conclusions

In this work, we established AS1411-functionalized liposomes encapsulating MTA2 siRNA for selective delivery to pancreatic cancer cells. The optimized construct exhibited a uniform particle size of ~180 nm, stable surface charge of −4.71 mV, and high siRNA encapsulation efficiency (>80%). In MIA PaCa-2 cells, AS1411-Lipm[siRNA] achieved ~60% reduction of MTA2 protein, accompanied by robust restoration of PTEN expression and ~50% suppression of AKT phosphorylation. These molecular changes resulted in ~50% decrease in cell viability, ~35% increase in apoptosis, and ~75% reduction in wound closure, whereas nucleolin-deficient L929 fibroblasts showed negligible responses. Further studies in three-dimensional cultures and animal models will be essential to validate the observed selectivity and signaling modulation in more complex biological systems. AS1411-Lipm[siRNA] demonstrated gene-selective silencing with tumor-cell specificity in PDAC, supporting its translational potential as a precise platform for RNAi-based intervention.

## Data Availability

Data that support the findings of this study are available within the article and Supplementary Material.

## Supplementary Materials

The supporting information can be downloaded at https://www.mdpi.com/article/10.3390/ijms26178467/s1.

## Abbreviations

The following abbreviations are used in this manuscript:

AS1411-Lipm[siRNA]	AS1411 aptamer-conjugated liposomes encapsulating MTA2 siRNA
CCKBR	Cholecystokinin B receptor
DAPI	4′,6-Diamidino-2-phenylindole
DLS	Dynamic light scattering
DOPE	1,2-Dioleoyl-sn-glycero-3-phosphoethanolamine
DOTAP	1,2-Dioleoyl-3-trimethylammonium-propane
ELS	Enhanced permeability and retention
Kd	Dissociation constant
Lipm	Naked liposomes
Lipm[SC]	Scrambled double-stranded RNA-encapsulated liposomes
Lipm[siRNA]	MTA2 siRNA liposomes
MFI	Mean fluorescence intensity
MMP14	Matrix metalloproteinase 14
MTA2	Metastasis tumor-associated protein 2
N/P ratio	Nitrogen-to-phosphate molar ratio
NCL	Nucleolin
PDAC	Pancreatic ductal adenocarcinoma
PDI	Polydispersity index
PEG-DSPE2000	1,2-Distearoyl-sn-glycero-3-phosphoethanolamine–polyethylene glycol 2000
PFA	Paraformaldehyde
PTEN	Phosphatase and tensin homolog
qRT-PCR	Quantitative reverse transcription polymerase chain reaction
siRNA	Small interfering RNA
TEM	Transmission electron microscopy
TfR1	Transferrin receptor 1
