# Supplementary material for: AS1411 Aptamer-Conjugated Liposomal siRNA Targeting MTA2 Suppresses PI3K/AKT Signaling in Pancreatic Cancer Cells
Source: Int J Mol Sci. 2025 Aug 30;26(17):8467. doi: 10.3390/ijms26178467 (PMC12428796; doi:10.3390/ijms26178467)
Supplement: Supplementary file 1 [file ijms-26-08467-s001.zip › ijms-3842569-supplementary.pdf]

Supplementary Materials

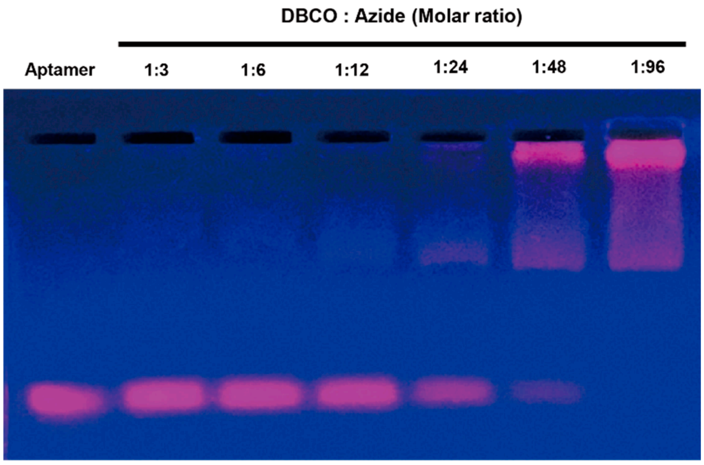

Figure S1. Conjugation of DBCO–AS1411 aptamer to azide-functionalized micelles at various molar ratios. DBCO–AS1411 aptamer and azide–micelles were mixed at molar ratios of 1:3, 1:6, 1:12, 1:24, 1:48, and 1:96, followed by incubation at 4 °C for 12 h to allow click conjugation. The reaction products were analyzed by 1% agarose gel electrophoresis pre-stained with GelRed. The disappearance of the free aptamer band was observed at a DBCO:azide ratio of 1:96, indicating complete conjugation. This ratio was selected as the optimized condition.

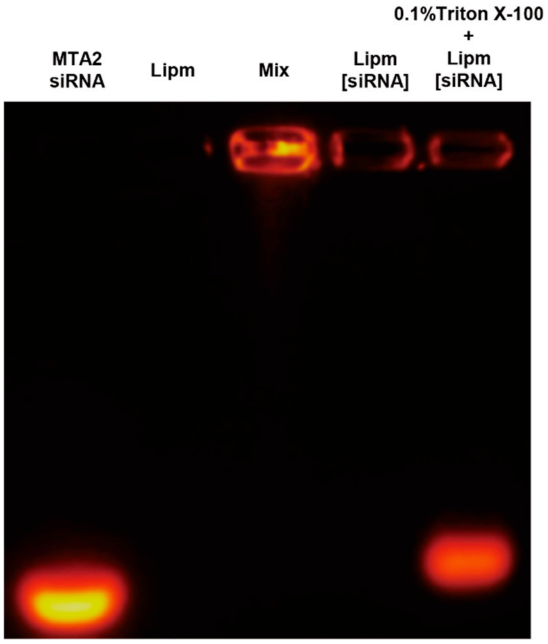

Figure S2. Evaluation of siRNA loading into Lipm at an N/P ratio of 4. FAM-labeled MTA2 siRNA was mixed with liposomes or encapsulated via film hydration at an N/P ratio of 4. Samples were analyzed by 1% agarose gel electrophoresis pre-stained with GelRed. In the third lane (Mix), where siRNA and liposomes were simply mixed, a strong fluorescent band was retained in the well, indicating surface association of negatively charged siRNA with the positively charged liposomal surface. In contrast, Lipm[siRNA] displayed no detectable siRNA band, suggesting that siRNA was encapsulated within the liposomes. Upon treatment with 0.1% Triton X-100, siRNA was released and migrated into the gel, confirming successful internal encapsulation.

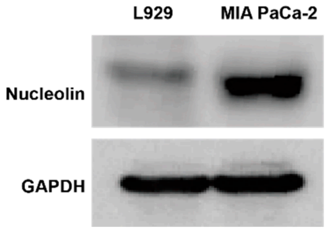

Figure S3. Western blot analysis of nucleolin expression in L929 and MIA PaCa-2 cells. Cell lysates from L929 and MIA PaCa-2 cells were subjected to western blotting using an anti-nucleolin antibody. Nucleolin expression was barely detectable in L929 cells, whereas strong expression was observed in MIA PaCa-2 cells, confirming that these cell lines can be classified as nucleolin-negative and nucleolin-positive, respectively.

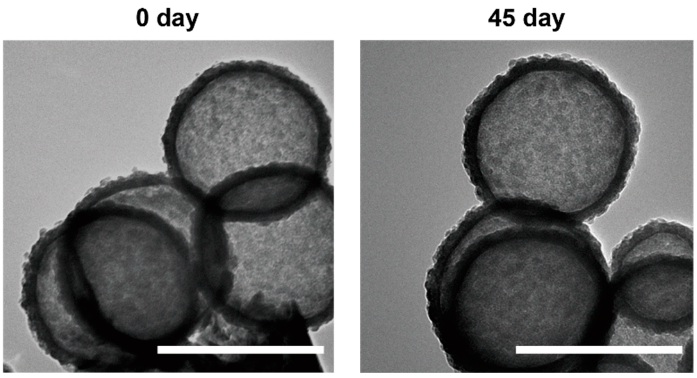

Figure S4. Morphology of AS1411-Lipm[siRNA] immediately after preparation (Day 0) and after 45 days of storage at 4 °C, visualized by transmission electron microscopy (TEM). The spherical structure was preserved without noticeable disruption, indicating structural stability of AS1411-Lipm[siRNA] under 4 °C conditions. Scale bar = 200 nm.
